# Valproate use in women aged 15–44 years: an observational study in general practice

**DOI:** 10.3399/BJGPO.2020.0104

**Published:** 2021-03-31

**Authors:** Samantha J Beardsley, Isabel Dostal, James Cole, Ana Gutierrez, John Robson

**Affiliations:** 1 Barts Health NHS Trust, The Royal London Hospital, London, UK; 2 Institute of Population Health Sciences, Queen Mary University of London, London, UK; 3 Institute of Population Health Sciences, Queen Mary University of London, London, UK; 4 Institute of Population Health Sciences, Queen Mary University of London, London, UK

**Keywords:** primary health care, valproate, women, adverse drug related event, pregnancy

## Abstract

**Background:**

Valproate is a known teratogen. In April 2018, the Medicines and Healthcare products Regulatory Agency (MHRA) restricted its use in women and banned use in pregnancy, except for epilepsy with no other effective treatment. To date, there is limited information on valproate prescribing within primary care.

**Aim:**

To characterise valproate prescribing to women of childbearing age, recorded advice or GP prescribed contraception, and recorded pregnancies.

**Design & setting:**

A cross-sectional study of patients from all 141 general practices across three clinical commissioning groups (CCGs) in East London.

**Method:**

Women aged 15–44 years prescribed valproate between 1 October 2017 and 1 January 2020 were included. Exclusion criteria were early menopause, sterilisation procedures, or hysterectomy. Pseudonymised data on valproate indication, pregnancy, pre-conception, and contraception advice were retrospectively extracted from general practice consultation data. Data were analysed by quarter using univariate statistics.

**Results:**

Of the total 1 042 463 registered patients, 344 women aged 15–44 years were prescribed valproate during the study period; 14 were excluded. There were 10 pregnancies during possible valproate exposure; one was terminated. During the study period, the number of women prescribed valproate significantly decreased (*P* = 0.003). The pregnancy rate decreased from 9.9/1000 on valproate before the MHRA April 2018 warning, to an average of 2.8/1000 afterwards. Recorded pre-conception and contraception advice increased by 79%, from 24% to 43%, of women prescribed valproate.

**Conclusion:**

With continued pregnancies in women aged 15–44 years prescribed valproate, patient education and foetal outcomes remain ongoing concerns. Further improvements are needed to ensure women make informed reproductive choices and safeguard future pregnancies from valproate exposure.

## How this fits in

Valproate is a known teratogen causing physical abnormalities and is also associated with neurodevelopmental delay, including autism. Recent MHRA guidance restricts the use of valproate in women with reproductive potential. This study shows an increase in patient valproate education and a reduction in valproate prescribing, but ongoing pregnancies within this group highlight continuing concerns about valproate exposure in pregnancy. A need to improve delivery of high quality patient education is demonstrated to enable women to make informed reproductive choices and safeguard future pregnancies against valproate exposure.

## Introduction

Valproate is an antiepileptic drug (AED) and mood stabiliser licensed in the UK for use in the treatment of all forms of epilepsy, mania in bipolar affective disorder, and migraine prophylaxis.^1^ Valproate is a known teratogen. The risks of valproate use in pregnancy are well documented with wide-ranging effects of in utero valproate exposure, including congenital malformations and neurodevelopmental delay.^2,3^

Of the AEDs, valproate has been consistently highlighted as having the highest rate of congenital malformations.^2,4^ Based on two major meta-analyses of data from studies worldwide, the rate of major congenital malformations in babies born to mothers taking valproate monotherapy during pregnancy has been estimated to be 11% compared with 3% in women without epilepsy.^2,3^

Beyond birth, there is consistent evidence that valproate use during pregnancy is associated with persistently impaired neurodevelopment, which affects 30%–40% of children exposed to valproate during this time.^4–7^ The effects are varied and include four times increased risk of psychomotor delay, seven times increased risk of cognitive development delay, and eight times increased risk of language delay.^8^ There are strong associations with the development of autism where risk is estimated to be increased up to 17 times.^3,8^ In utero valproate exposure is also associated with a significant decrease in intelligent quotient (IQ) of around 8 IQ points.^3^ This reduction in IQ alone is likely sufficient to affect education and lifelong occupation.^3^ Most congenital birth defects and neurodevelopmental complications arising from in utero valproate use will have a lifetime financial, medical, and social care burden for affected families and the state.^9^

In response to the accumulating body of evidence of valproate risk, as well as pressure from patient advocate groups, there have been several stepwise changes in public policy and clinical guidelines. In the UK, the MHRA issued a press release in April 2018 restricting the use of valproate in women of childbearing potential unless they are on a pregnancy prevention programme.^10^ The pregnancy prevention programme aims to enable women and their clinicians to make informed decisions about their care by providing information about the risks of pregnancy and providing highly effective contraception, including long-acting reversible contraception (LARC) methods such as intrauterine devices and the progesterone-only implant.^11^ Overall trends since then suggest a substantial reduction in valproate prescriptions in women of childbearing age,^12^ but little is known about the extent to which the 2018 MHRA guidance has been implemented.

This study aimed to characterise: valproate prescribing to women of childbearing age, recorded advice or contraception prescribed by GPs, and recorded pregnancies between 2017 and 2020 across three CCG areas of East London.

## Method

This cross-sectional study included de-identified general practice data from all 141 GP practices in three adjacent inner-urban CCGs in East London (City and Hackney, Tower Hamlets, and Newham) between 1 October 2017 and 1 January 2020. These CCG areas are among the most ethnically diverse and deprived in both London and the UK. These GP practices all use EMIS Web (Egton Medical Information Systems) as an electronic health record to document patient interactions and prescribing. De-identified patient data for the relevant study items were centrally extracted and securely stored by the Clinical Effectiveness Group (CEG), Queen Mary University of London. The types of data accessed by the CEG and the information sharing role of the CEG has been described elsewhere.^13^

De-identified data were retrospectively extracted for the start of each quarter between 1 October 2017 and 1 January 2020 to cover the 6 months before the release of the MHRA guidance in April 2018 and the time until this study (that is, until data extraction). The cohort were women aged 15–44 years currently registered at the 141 practices who were prescribed valproate in the past 3 months, before the relevant start date in the quarter. Valproate prescriptions included all generic preparations of valproate, sodium valproate, semisodium valproate, and all brand names available in the UK (Epilim Chrono, Epival, Depakote, Episenta, and Convulex). Patients were excluded if they had previously undergone early menopause, sterilisation procedures, or hysterectomy. Pregnancy was inferred through an algorithm combininga number of Read codes related to pregnancy and used in the national immunisation pregnancy codeset.^14^ Data extraction took place in February 2020; the database was not updated during this time.

Read codes of interest were identified relating to inclusion (women aged 15–44 years prescribed valproate) and exclusion criteria (early menopause, sterilisation, and hysterectomy), pre-conception advice, contraception advice, provided or prescribed contraception, and long-term problems as the likely indication for valproate prescription (See Supplementary Table S1).

Data for these Read codes were extracted for specific timeframes, relative to the Read code date of the valproate prescription, within the quarter (see Supplementary Table S1). Duplicates were removed with the first occurrence being used for analysis. All data were analysed using R (version 3.6.2), including linear regression to compare outcomes and assess statistical significance. Missing data were interpreted as that outcome not being true for that patient at that time, as recorded by the GP. All patients were assigned a pseudonymised identifier as part of the extraction process. This work was conducted according to STROBE guidelines on reporting observational studies.^15^

## Results

Quarterly data were extracted between 1 October 2017 and 1 January 2020 for the three CCG areas with a total of 1 042 463 registered patients in 2018 (City and Hackney, *n* = 318 637; Newham, *n* = 398 907; and Tower Hamlets, *n* = 324 919). There were a total of 344 women aged 15–44 years prescribed valproate, of whom 14 patients were excluded (early menopause, *n* = 1; sterilisation procedures, *n* = 10; or hysterectomy, *n* = 3) to give a final sample size of 330. The median age of the sample was 34 years (interquartile range 27–41 years) (Table 1). The ethnic group of the sample was predominantly White followed by South Asian.

**Table 1.** Demographic characteristics of 330 women aged 15–44 years prescribed sodium valproate between 1 October 2017 and 1 January 2020 in East London

Age, years^a^	***n* (%**)
15–19	38 (12)
20–24	22 (7)
25–29	54 (16)
30–34	69 (21)
35–39	60 (18)
≥40	87 (26)
**Ethnic group** ^a^	
White	150 (45)
Black	49 (15)
South Asian	87 (26)
Other	22 (7)
Unknown	22 (7)
**Received pre-conception or contraception advice** ^b^	151 (46)
**Prescribed contraception** ^b^	90 (27)
Prescribed LARC^c^	48 (53)
Prescribed non-LARC^c^	42 (47)
**Received advice or contraception** ^b^	191 (58)

^a^Data relate to most recent record for age and ethnic group. ^b^At least once during study period. ^c^Long-acting reversible contraception (LARC): copper intrauterine device, levonorgestrel 13.5 mg/19.5 mg/52 mg intrauterine system, and progestogen-only implant. Non-LARC contraception: combined hormonal contraception including combined contraceptive pill, transdermal patch and vaginal ring, progestogen-only pill, progestogen-only injectable depot medroxyprogesterone acetate, condoms, and diaphragms.

**Figure 1.**
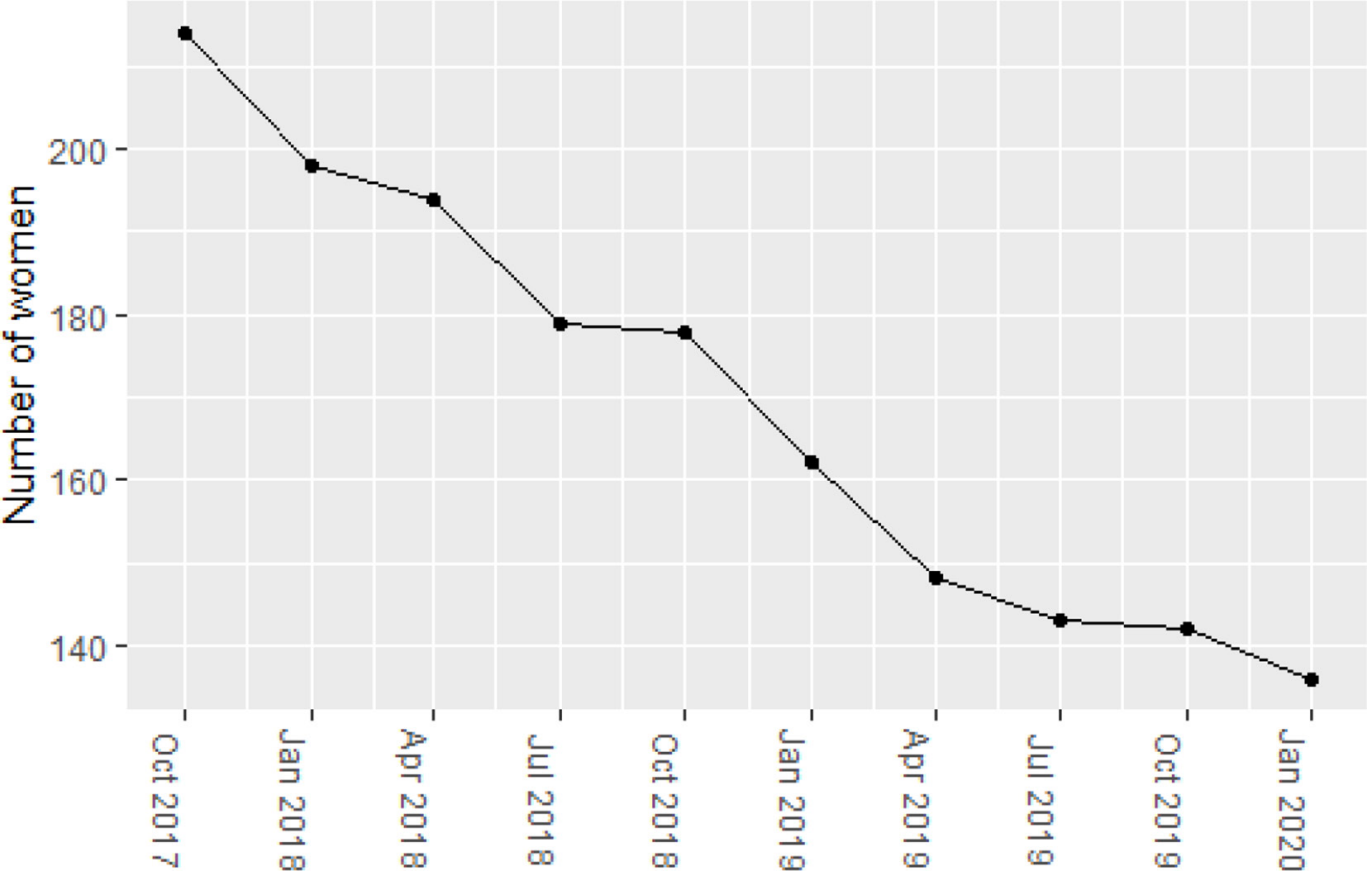
Number of women prescribed valproate between 2017 and 2020 in East London

Of the total 330 unique individuals, 63 were prescribed valproate at every quarterly time point in the study period. Over this time, the total number of women prescribed valproate decreased by 36% from 214 in October 2017 to 136 in January 2020 (Figure 1). This decrease in valproate prescribing before and after the MHRA warning in April 2018 was statistically significant (*P* = 0.003).

In total, there were 10 pregnancies in nine women prescribed valproate during the study period, one of which was terminated (Table 2). The pregnancy rate (defined as the number of pregnancies in the year divided by the number of women on valproate x 1000) averaged 9.9/1000 in the three quarters before the MHRA announcement in April 2018 to an average of 2.8/1000 in the quarters after April 2018, which was a decrease of 71%. (Table 2). The median age of these women was 30 years (interquartile range 28–32 years). Of these women, 89% (*n* = 9) were recorded as having epilepsy, 22% (*n* = 2) were recorded as having bipolar affective disorder, and 11% (*n* = 1) were on the learning disability register. In terms of ethnic group, 40% (*n* = 4) of these women were in White and 50% (*n* = 5) were in Black, South Asian, and Other ethnic groups (missing data *n* = 1). Two of these women were recorded as having received pre-conception advice; however, only one of these was before the recorded pregnancy. Four of these women were recorded as having received contraception advice; this was recorded on two occasions before pregnancy (data not shown).

**Table 2.** Pregnancy and pregnancy rates by quarter between 1 October 2017 and 1 January 2020 in East London

	Quarter
10/17	01/18	04/18	07/18	10/18	01/19	04/19	07/19	10/19	01/20
Total, *n*	214	198	194	179	178	162	148	143	142	136
Pregnancies, *n* ^a^	4	1	1	0	0	1	0	1	0	1
Pregnancy rate (average per 1000 population)	9.9			2.8						

^a^New pregnancies in this quarter; corrected for one termination or miscarriage.

Indications for valproate for these 330 individuals is shown in Table 3. The most common indication for women prescribed valproate, at both the start and end of the study period, was epilepsy (65%; *n* = 139 and *n* = 88, respectively) followed by bipolar disorder (14% and 15%; *n* = 31 and *n* = 20, respectively). The distribution of indications was largely unchanged at the end of the study period despite an overall reduction in the number of women prescribed valproate.

**Table 3.** Indication for valproate for women included in the first and last quarters of the study period

Valproateindication^**a**^	Dataextraction from 1 Oct 2017	Data extraction from 1 Jan 2020
*n* (%)	*n* (%)
Epilepsy	139 (65)	88 (65)
Bipolar disorder	31 (14)	20 (15)
Migraine	4 (2)	2 (1)
Learning difficulties	5 (2)	5 (4)
Other or unknown	35 (16)	21 (15)
Total patients	214 (100)	136 (100)

^a^Likely indication inferred from recorded long-term conditions on electronic health record.

**Figure 2.**
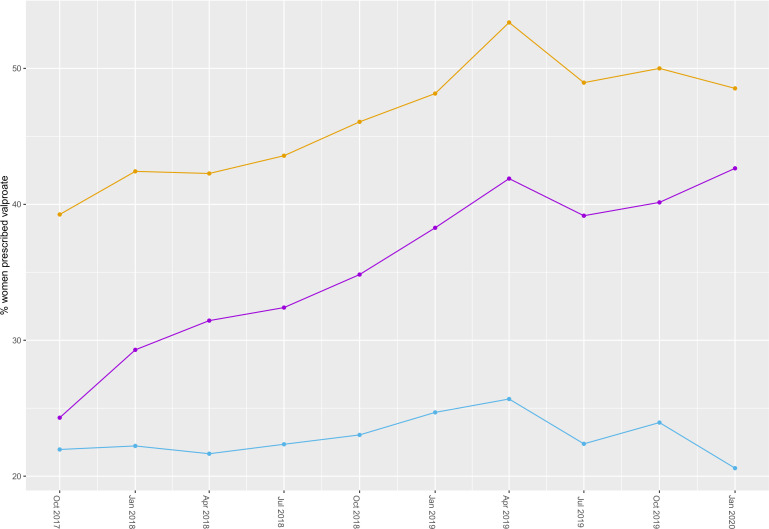
Change in pre-conception or contraception advice (purple), prescribed contraception (blue), and women receiving advice and/or contraception (orange), normalised to the number of women prescribed valproate

The proportion of women prescribed valproate receiving pre-conception advice or advice on contraception increased by 79% during the study period from 24% (*n* = 52/214 women) to 43% (*n* = 58/136 women) (Figure 2). Recorded contraception prescribing rates stayed approximately constant at around 20%. At the beginning of the study period, 39% of women (*n* = 84/214) prescribed valproate were given advice and/or contraception, which had increased to 49% (*n* = 66/136) by the end of the study period.

## Discussion

### Summary

Ten pregnancies potentially exposed to valproate were identified between October 2017 and January 2020 in East London. Pre-conception or contraception advice was poorly recorded in the general practice record system before these pregnancies, with only one woman with pre-conception advice and two women with contraception or contraceptive advice recorded. This may be owing to a failure of appropriate counselling or a failure to record such information, or both. Such failures may limit the ability of women to make informed decisions about their treatment with valproate and about family planning.

Over the study period, there was an overall improvement in the recording of pre-conception and contraception advice to women of childbearing age prescribed valproate, which was in association with a 71% decrease in pregnancy rate in women of childbearing age prescribed valproate. This suggests the MHRA 2018 recommendations led to increased levels of recording and/or patient education, with more women making informed decisions about their ongoing care in relation to valproate use and pregnancy.

There was a statistically significant decrease in the number of women prescribed valproate before, compared with after the April 2018 MHRA warning. This decrease was most pronounced in the year following the MHRA announcement. However, the decline has slowed since then and a valproate prescription in general practice continues to pose substantial risks to pregnancies in these women.

The MHRA guidance bans the use of valproate for women during pregnancy for psychiatric and all other indications except for epilepsy if it is the only option, and restricts its use more generally unless combined with effective contraception and a pregnancy prevention plan.^10^ The majority of the 10 at-risk pregnancies identified were in women with epilepsy, which may reflect that there was no alternative treatment. However, despite an overall reduction in the number of women prescribed valproate, the proportion of women prescribed valproate for indications other than epilepsy remained largely unchanged between the start and end of the study period; for example, a number of women were still prescribed valproate for bipolar affective disorder or learning disabilities where alternative treatments were more appropriate. Changing prescribing for these women will reduce the risk of further valproate-exposed pregnancies.

### Strengths and limitations

These data are from a large number of urban GP practices that are located in areas of high ethnic diversity and deprivation, which are factors well documented to contribute to health inequalities.^16^ However, contraceptive and pre-pregnancy advice are activities undertaken by all GPs and there is no reason to assume that those in the study locality differed in their advice and prescription from any other area.

Prescribing for long-term conditions is undertaken almost entirely by GPs for whom the electronic health record is an accurate record. However, not all prescribed medication may actually be dispensed or used by the patient, and it is possible that use of prescribed valproate by patients was stopped before or at some time during pregnancy. This study is only able to identify where GPs have recorded that pre-conception and contraception advice was given, so these figures may underestimate the true numbers of women who received such advice from other sources. Some women are likely to have contraception managed by third-party clinics or were using other methods including rhythm methods, condoms or diaphragms, and partner vasectomy. These are all likely to be poorly recorded in a woman’s record but may be more likely to be subsumed in the coding for ‘contraception advice given’.

Current pregnancy was inferred in this study, using codes indicating a current pregnancy, including pregnancy, antenatal care, and a duration of 9 months from the earliest record of pregnancy. It is possible that some women were not pregnant at a time of overlapping valproate prescription. The number of pregnancies in this study was small and a larger scale study with mother and baby linkage is required to assess the effects of valproate on foetal outcomes.

### Comparison with existing literature

The authors are not aware of any recent studies of valproate use that have considered contraceptive and pregnancy outcomes in UK general practice since the MHRA warning. Despite the recent input from the MHRA, there have been concerns for many years about a lack of patient education surrounding valproate use in women of childbearing potential.^7,10,17^ A 2017 survey of women of childbearing potential prescribed valproate for epilepsy conducted by the Epilepsy Action, Epilepsy Society, and Young Epilepsy suggests around 70% of women surveyed had not received information about changes in advice about the pregnancy-associated risk.^18^ Further to the 2018 MHRA guidance, joint guidelines from the Royal College of General Practitioners and Royal College of Physicians have provided advice to clinicians on how to broach, record, and manage these conversations with women of childbearing age.^11^ GP recording of the pregnancy prevention programme is in its infancy and there is currently no way to easily electronically access or record the MHRA consent form signed by patient and specialist.

The Neurodevelopmental Effects of Antiepileptic Drugs study indicated that valproate use in pregnancy in the US and UK reduced between 1999 and 2007.^19^ More recently, primary care data collected between 2010 and 2019 in the UK suggest an overall decline in the initiation of valproate prescription for females aged <45 years.^12^ In particular, the authors noted an 80% decrease in initiation of valproate in females in the first half of 2019 compared with the first half of 2010. This trend was noted across all indications (epilepsy, bipolar disorder, migraine, and unknown), with epilepsy being the most common indication for valproate use.^12^ The Cumberlege report published after this study has underlined the substantial risks of valproate prescribing, and the need to improve safety and monitoring of outcomes.^20,21^

### Implications for research and practice

There is currently no standard pathway in the UK for following up babies born to mothers who have taken valproate during pregnancy.^11^ Given that some neurodevelopmental issues may not arise for several years, in utero medication exposure may be overlooked as a cause. This study has identified nine children that might benefit from follow-up in this locality. At a research level, this could be done through de-identified linkage of primary and secondary care information with an appropriately large sample size. There are also plans to create a valproate register for children of mothers taking valproate during pregnancy.^11^

For clinicians, this work demonstrates the need to improve patient education for women of childbearing potential prescribed valproate. At a practice level, accurate recording of patient encounters is required to reflect the quality of care that is being provided.

Valproate prescribing to women aged <45 years has significantly decreased since the April 2018 MHRA warning. However, this study shows that despite the MHRA recommendations, valproate prescription, in association with a lack of recorded advice as well as continuing prescription outside of MHRA guidance, may continue to place women and their children at risk of exposure during pregnancy. This demonstrates the need for improved patient education and recording of patient encounters to ensure that women receive high quality care to inform reproductive choices.
